# Effects of Maturation on Plantar Flexor Activity and Achilles Tendon Stiffness in Vertical Jumping: Sex Differences

**DOI:** 10.3390/sports12100284

**Published:** 2024-10-21

**Authors:** Zacharoula Paschaleri, Georgios Chalatzoglidis, Theodoros Kannas, Fotini Arabatzi

**Affiliations:** Department of Physical Education and Sport Science, Laboratory of Neuromechanics, Aristotle University of Thessaloniki, 62100 Serres, Greece; zpaschal@phed-sr.auth.gr (Z.P.); gchalatzo@phed-sr.auth.gr (G.C.); thkannas@phed-sr.auth.gr (T.K.)

**Keywords:** growth spurt, stretch-shortening cycle, tendon properties

## Abstract

The aim of this study was to investigate the effect of maturation on vertical jumping performance, in adolescent boys and girls, concerning plantar flexor activity and Achilles tendon (AT) stiffness. Thirty-nine adolescents were tested in a counter-movement jump (CMJ) at three different time points: 18 and 9 months before peak height velocity (PHV) and at PHV. The EMG activity of the medialis gastrocnemius (MG) and tibialis anterior (TA) muscles was evaluated, in relation to jump height. Boys showed higher jumping ability and AT stiffness than girls. Additionally, boys revealed increased eccentric (ecc) and concentric (con) MG activity, along with decreased ecc and con TA activity, near PHV. On the other hand, girls showed increased ecc and con TA/MG co-contraction compared to boys, mainly near PHV. In conclusion, a different mechanism of vertical jumping performance is adopted between early adolescent boys and girls. Nevertheless, no notable alterations in jumping capability were detected over time, indicating that the maturation process does not influence stretch-shortening cycle (SSC) performance.

## 1. Introduction

Jumping and hopping are SSC applications and common actions during adolescent life, not only in daily activities but also in competitive sports [1]. It is well established that muscle force and tendon stiffness affect performance in jumping [2]. In late adolescents and adults, the presence of basal anabolic hormones causes a development in muscle size, simultaneously with an increase in muscle strength. However, in preadolescents, the gains in muscle strength are caused by neural adaptations, with long-term strength training having questionable effects on children’s muscle hypertrophy, primarily in certain muscle groups [3]. However, the mechanisms linked to force production and, consequently, to jumping performance in early adolescents, around the age of peak height velocity (PHV), are not well studied. This lack of evidence highlights the necessity for further research to better understand the interaction between muscle hypertrophy and neural adaptations in enhancing SSC performance during this critical developmental period.

Previous studies suggest that both maturation and neuromuscular training may increase motor-unit activation, leading to improved SSC function. As children grow, their concentric and eccentric force capabilities increase, possibly resulting in greater capability during SSC activities, like sprinting and jumping [1,2]. Although increases in muscle mass could enable more force and power production during SSC performance [1,2], it seems that neural adaptations, such as improved motor unit recruitment, increased pre-activation, and decreased agonist–antagonist co-contraction precede the adaptation in muscle level, playing a crucial role in SSC performance. Therefore, while muscle hypertrophy, as a direct result of maturation in children, is certain, adaptations in SSC function might still be due to increased motor unit activation and improved neuromuscular efficiency rather than muscle hypertrophy [4,5,6].

Although the mechanical properties of the muscle–tendon system play a crucial role in SSC control, most of these alterations occur during puberty. Thus, the contribution of the neuromuscular system to the SSC is a vital indicator of final performance [7] before PHV. Recent findings suggest that co-contraction around the ankle joint is higher in children and decreases with maturation. As previously reported, adolescent maturation seemed to cause a decrease in the co-contraction of knee extensors during maximal isometric contractions [8]. Agonist–antagonist co-contraction of the lower limb enhances joint stiffness and stability, resulting in reduced eccentric loading during the stretch phase. These findings align with previous studies on pre-adolescents aged 9–11, indicating greater TA activity and less MG activity during vertical jumps compared to more mature adolescents and adults [9,10]. Moreover, increased antagonist activation and co-contraction lead to less efficient movement and reduced performance within the SSC. However, the electromyographic pattern of ankle joint muscles during vertical jumps in children around PHV has not been longitudinally investigated.

It has been shown that tendinous tissues transfer the produced muscle forces to the bones [11]. Additionally, due to their elasticity, tendons can affect the muscle’s power capability through the mechanism of the storage and release of strain elastic energy, a mechanism that affects efficiency in stretch-shortening exercises [12]. Around the age of PHV, children’s force capacity increases along with their body weight, causing a greater mechanical load on their tendons. On the other hand, the rapid rate of height increases affects or even disturbs the improvement of movement efficiency, which happens through adolescence. A recent longitudinal study showed that as children reach adolescence, the increase in muscle force also leads to greater stiffness in the AT. However, it has been suggested that around the age of PHV, the development of neural control in jumping and the potential occurrence of adolescent awkwardness contribute to a plateau in performance [13]. Neuromuscular and sensorimotor adaptations in structural and neural changes throughout the growth spurt could be the reason for these disturbances, but this is not fully understood yet.

Sex differences in AT stiffness and its effect on jumping performance have revealed inconclusive results [7]. Recent research revealed a greater level of stiffness for males compared to females in the AT, mainly due to differences in tendon dimensions, while others have suggested no differences and claimed that methodological differences were the reason for these conflicting findings. A recent longitudinal study in adolescents before the age of PHV has demonstrated a more rapid increase in AT stiffness in boys than girls, linked to increased force capability [14]. However, a smaller longitudinal study of a six-month duration revealed no differences at the same biological age between sexes [15]. Concerning CMJ performance, a recent study of 720 athletes from many sports found higher jumping performance for boys/men compared to girls/women in a cohort of late adolescents and adults [16]. However, as far as we know, there are no longitudinal data on the influence of AT stiffness and plantar flexor force on CMJ performance in adolescent boys and girls during maturation.

It was postulated that alterations in AT stiffness, in addition to longitudinal modifications in force generation by the plantar flexors, would influence vertical jump performance in disparate ways between boys and girls during the adolescent growth spurt. Therefore, the objective of this study was to examine the impact of maturation on the development of stiffness and muscle activity in the plantar flexors, and thus, to investigate how performance in vertical jumping is regulated in adolescent boys and girls at the age of PHV.

## 2. Materials and Methods

### 2.1. Participants

Twenty boys (aged 12.5 ± 0.29) and 18 girls (aged 10.5 ± 0.32) participated in this study. Children were randomly selected from primary and high schools of Serres, Greece. The only inclusion criteria pertained to age, with girls being 10.0–10.5 years old and boys 12.0–12.5 at the beginning of the study. This age difference between boys and girls was in accordance with previous findings that girls enter puberty two years earlier than boys, at about the age of 10.5 years old [17]. The participants in the present study were involved in track-and-field sports activities, and it was assumed that the effect of training was approximately equivalent for all of them in terms of their performance in vertical jumping. For every participant, we estimated maturity offset (time relative to PHV) using a sex-specific multiple regression equation [18]. All children were required to be free from any neurological or orthopedic conditions. The exclusion criteria included the presence of chronic illness, recent injuries, or any medical conditions that could affect neuromuscular function or musculoskeletal integrity. Parents or guardians signed a consent form. The research protocol and measurement methods received approval from the Local Ethics Research Committee (ERC-009/2020). This study was conducted in compliance with the ethical guidelines and recommendations governing clinical and field science research [19].

### 2.2. Overall Study Procedures

CMJ measurements took place in the same laboratory and at same time of the day for each of the three measurements (18 and 9 months prior to PHV, as well as at PHV). Participants performed a 10 min warm-up session that included jogging and standing quadriceps-, calf-, and TA-stretching exercises. Then, they performed two familiarization trials of the CMJ on a Kistler force platform (9281 CA, Kistler Instruments Ltd., Winterthur, Switzerland) and two more vertical jumps, with a 2′-break between the trials. The trial with the best jump height was analyzed.

### 2.3. CMJ Measurement

The jump began from a standing position, with participants’ hands placed on their iliac crest. The children were instructed to flex their knees to approximately 90° (checked by visual observation) before rapid extension and take-off with the knee angle extended at 180°. Landing was on the toes with both feet parallel and apart. If these criteria were not met, the jump was performed again.

### 2.4. EMG Measurements

The muscle activity of the MG and TA during the eccentric and concentric phases of the jump was measured using bipolar surface EMG electrodes (Motion Control, Biomed Products Inc., Truckee, CA, USA) voltage range: 64–612 V, interfaced with a 16-channel analog amplifier (sampling frequency 1000 Hz, CMRR 100 db at 50/60 Hz, bandwidth 8500 Hz, gain 400). The preparation of the skin and placement of the sensors followed the recommendations of the SENIAM project [20]. All signals were synchronized and converted from analog to digital using a Biopac MP150 unit (Biopac Systems Inc., Goleta, CA, USA) with a 1 kHz sampling frequency at 16 bits. The RMS values obtained during the CMJ were normalized with respect to the maximal values derived from the same test (normEMG). Coactivation between the TA and MG muscles during the eccentric and concentric phases of the jump was calculated using the following equation: (normEMG of TA/normEMG of MG) × 100 = (coactivation index; CI). OT Biolab+ (v. 1.5.80 OT Bioelettronica, Torino, Italy) software was used to synchronize the force and EMG data, while the signal processing and the calculation of the statistical values were conducted using MatLab (v.17, The Mathworks Inc., Natick, MA, USA).

### 2.5. Methodology for Determining Achilles Tendon Moment Arm and Tendon Force

Participants were positioned in a prone posture on a dynamometer bench (Cybex Humac Norm, CSMI, Stoughton, MA, USA) with their knees fully extended. The right foot was maintained at a 90-degree angle relative to the tibia, ensuring full knee extension, and securely attached to the dynamometer’s footplate. Measurements of torque were taken at the neutral ankle position (0 degrees). The moment arm (MA) of the AT was calculated using the excursion method, whereby the tibia was considered to represent the stationary segment and the calcaneus the entire rotating foot. The distance between the attachment points of the AT on the calcaneus at the initial and final positions of the foot in rotation (−20° → +20°) was digitized and considered to represent the excursion of the AT, as detailed in earlier studies [21]. The MA is defined as the perpendicular distance from the center of rotation to the line of action of the AT. A previously described method was used to detect heel movement during muscle contraction [22] and to correct measurement errors caused by the effects of ankle joint rotation.

Tendon force was estimated using the equation F = M/d, where F represents tendon force, M is the plantar flexion moment, and d is the length of the AT moment arm. The antagonistic moment generated by the TA was calculated using methods outlined in previous research whereby the moment and EMG signals from the triceps surae and tibialis anterior were measured. The RMS differences between plantar flexor moments were calculated considering the antagonistic co-contraction estimated at the same ankle angle at which the maximal plantarflexion moment was achieved [23]. The maximal plantar flexor moment was adjusted to include these antagonistic moment values, resulting in the total plantar flexor moment being the sum of the resultant joint moment and the antagonistic moment. Stiffness (k), in units of N/mm, was determined as the slope of the linear segment in the force (F)–elongation (ΔL) relationship. This relationship was constructed by combining force–time data with MTJ position–time data for each ramped isometric plantarflexion measurement. The data for muscle force (N) and MTJ length change (mm) were synchronized, and the slope of the F–ΔL relationship was calculated using the formula: k = dF/dL, as previously described [24].

### 2.6. Statistical Analyses

A repeated measures ANOVA was conducted to examine the effects of time and gender on dependent variables related to muscle activity, coactivation, stiffness, and jump height using SPSS statistical software (v.25.0, SPSS Inc., Chicago, IL, USA). A between-subjects analysis of variance (ANOVA) was conducted to examine the effect of gender on the dependent variables. The dependent variables included the relative eccentric and concentric contraction root mean square (RMS) for the MG (eccRMSMG, conRMSMG) and TA (eccRMSTA, conRMSTA) muscles, relative co-contraction (ecccoRMS, concoRMS), AT stiffness, jump height (height), and relative jump height (relheight). A pairwise comparison was conducted using Bonferroni confidence interval adjustment for each sex to assess the differences between different time points in the height, relheight, AT stiffness, and EMG variables: eccRMSMG, conRMSMG, eccRMSTA, conRMSTA, ecccoRMS and concoRMS. Statistical significance was accepted at a *p*-value < 0.05.

## 3. Results

The between-subjects analysis revealed a statistically significant difference between boys and girls in the variables eccRMSTA, eccRMSMG, conRMSMG, stiffness, height, and relheight for the time interval between 18 months before and the onset of PHV (Table 1).

### 3.1. Jumping Height

No significant differences were found between any time points in height or relative height for either boys or girls. CMJ height was higher in boys at Time 1 (M = 5.848, *p* = 0.023) and Time 2 (M = 9.631, *p* < 0.001).

### 3.2. AT Stiffness

Effect of maturation in boys: There was a significant increase in stiffness from Time 1 to Time 2 (M = 249.128, SE = 27.629, *p* < 0.001, 95% CI [179.750, 318.506]). A significant increase was also observed from Time 1 to Time 3 (M = 242.978, SE = 20.458, *p* < 0.001, [191.608, 294.348]). No significant difference was found between Time 2 and Time 3 (M = 6.150, SE = 37.345, *p* = 1.000).

**Figure 1:**
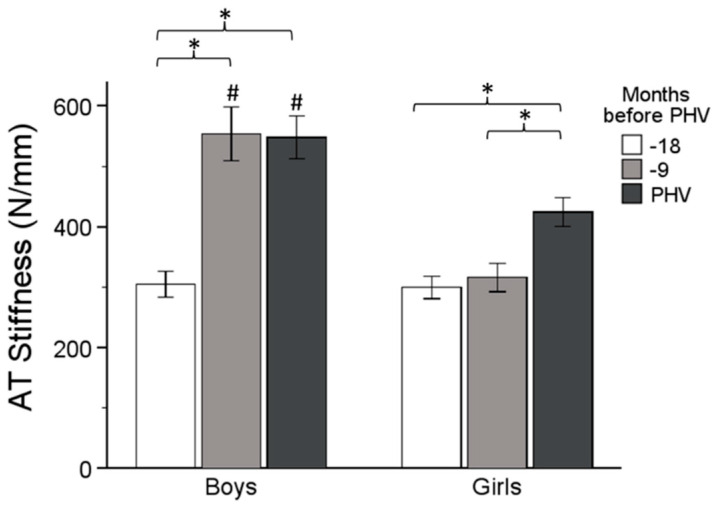
Achilles tendon stiffness changes up to PHV. Significant difference between time points (*), and between boys and girls at similar timepoints (#).

Effect of maturation in girls: A significant difference was found between Time 1 and Time 3, with an increase in stiffness (M = 124.740, SE = 21.564, *p* < 0.001, [70.592, 178.888]). A significant difference was also found between Time 2 and Time 3 (M = 107.892, SE = 39.365, *p* = 0.028, [9.045, 206.739]). No significant difference was found between Time 1 and Time 2 (M = 16.848, SE = 29.124, *p* = 1.000) (Figure 1).

Effect of sex: Boys had greater AT Stiffness than girls at Time 2 (M = 237.933, *p* < 0.001) and Time 3 (M = 123.891, *p* < 0.001) (Figure 1).

### 3.3. EMG during CMJ

**Figure 2:**
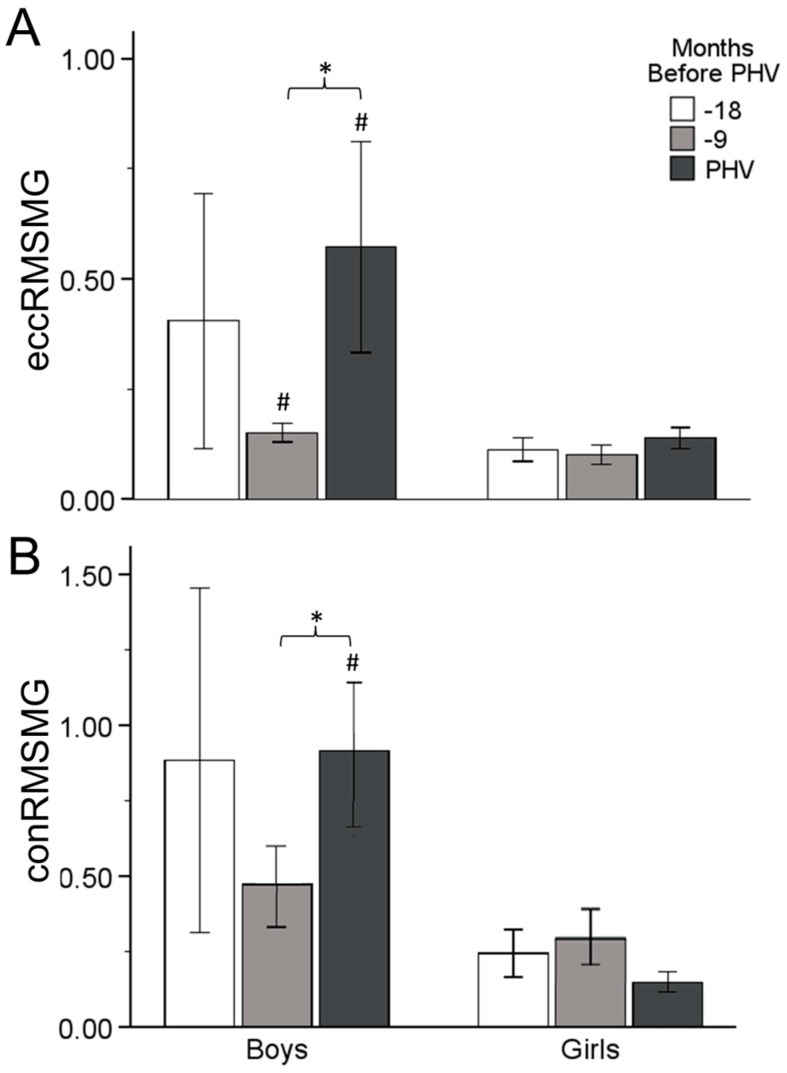
Relative eccentric (**A**) and concentric (**B**) EMG RMS of MG changes up to PHV. Significant difference between time points (*), and between boys and girls at similar timepoints (#).

A significant difference was found between Time 2 and Time 3, with an increase in eccRMSMG (M = 0.421, SE = 0.117, *p* = 0.003, [0.128, 0.715]) in boys. No differences were found between any time points in eccRMSMG in girls. Boys had higher eccRMSMG than girls at Time 2 (M = 0.049, *p* = 0.025) and Time 3 (M = 0.433, *p* = 0.018) (Figure 2).

A difference was also observed between Time 2 and Time 3, with an increase in conRMSMG (M = 0.443, SE = 0.108, *p* < 0.001, [0.171, 0.714]) in boys. No differences were found between any time point in eccRMSMG and conRMSMG in girls (Figure 2). However, boys had higher conRMSMG than girls at Time 3: M = 0.767, *p* = 0.011 (Figure 2). 

**Figure 3:**
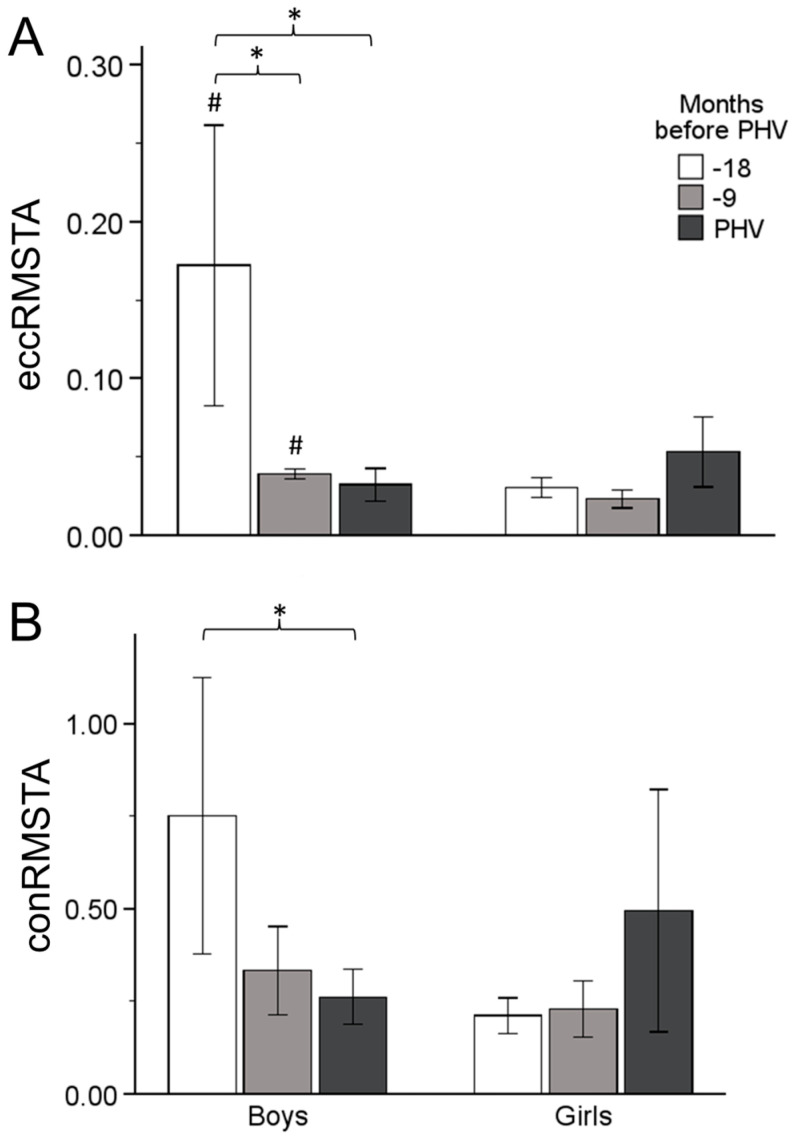
Relative eccentric (**A**) and concentric (**B**) EMG RMS of TA changes up to PHV. Significant difference between time points (*), and between boys and girls at similar timepoints (#).

There was a significant decrease in eccRMSTA from Time 1 to Time 2 (M = −1.329, SE = 0.451, *p* = 0.017, [−2.462, −0.196]) and from Time 1 to Time 3 (M = −1.397, SE = 0.477, *p* = 0.018, [−2.596, −0.199]) in boys, but there was no significant difference between Time 2 and Time 3 (M = 0.068, SE = 0.119, *p* = 1.000, [−0.232, 0.368]). There were no significant differences in eccRMSTA between any of the time points: Time 1 vs. Time 2: M = 0.072, SE = 0.476, *p* = 1.000; Time 1 vs. Time 3: M = −0.229, SE = 0.503, *p* = 1.000; Time 2 vs. Time 3: M = −0.301, SE = 0.126, *p* = 0.066 (Figure 3). Boys had higher scores than girls, with significant mean differences, at Time 1 (M = 1.418, *p* = 0.036) and Time 2 (M= 0.161, *p* = 0.001) (Figure 3).

A significant difference was found between Time 1 and Time 3, with a decrease in conRMSTA (M = −0.418, SE = 0.141, *p* = 0.016, [−0.772, −0.064]) in boys. No significant differences were found between any time points in conRMSTA (Figure 3).

**Figure 4:**
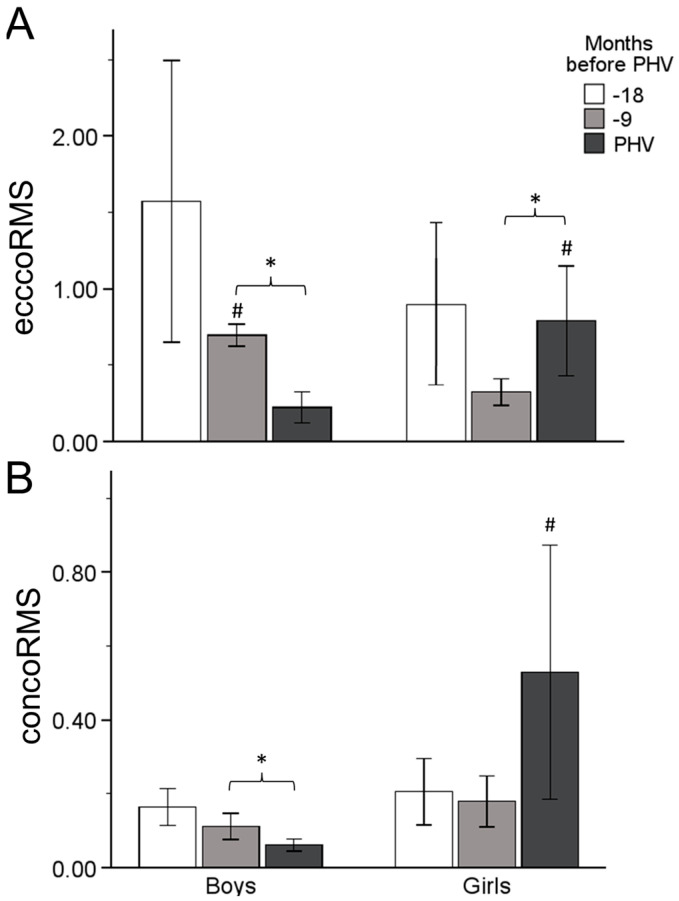
Relative eccentric (**A**) and concentric (**B**) EMG RMS of TA/MG co-contraction changes up to PHV. Significant difference between time points (*), and between boys and girls at similar timepoints (#).

There was a significant difference between Time 2 and Time 3, with a decrease in ecccoRMS (M = −473.685, SE = 167.655, *p* = 0.023, [−894.672, −52.697]) in boys. There was difference between Time 2 and Time 3, with an increase in ecccoRMS (M = 467.063, SE = 176.724, *p* = 0.036, [23.303, 910.823]) in girls. Significant differences were also found between boys and girls at Time 2 (M = 373.115, *p* < 0.001) and Time 3 (M = 567.633, *p* = 0.026). No differences were found between any time points in concoRMS for either boys or girls. However, boys had lower concoRMS scores compared to girls at Time 3: M = −467.839, *p* = 0.044 (Figure 4).

## 4. Discussion

In this study, it was hypothesized that there is an association between AT stiffness and jumping performance in early adolescent boys and girls and a different effect of maturation in the mechanism of vertical jumping between the two sexes. These results demonstrated that boys show greater jumping ability compared to girls at every time point of the maturation process. On the other hand, none of the sexes revealed significant changes in jumping ability over time, showing that the maturation process does not affect SSC performance, despite the alterations in tendon properties and muscle activation. Boys showed that AT stiffness increased in the period from 18 to 9 months, before PHV, while girls demonstrated a significant increase from 9 months to PHV. These changes were accompanied by changes in agonist and antagonist activation. Specifically, increased MG activity in boys and increased co-contraction in girls, mainly near PHV, lead to a different mechanism of jumping performance. Maturation affects the two sexes in a different way, with boys undergoing altered neural activation to keep up with their tendon properties, while girls adopt a mechanism combining the stiffer tendon with a stiffer joint to optimize jumping performance.

Despite the differences in tendon properties and neural activation between the two sexes, jumping height showed a plateau in time for both of them. Previous studies have shown that despite a significant increase in jumping ability from age 8 to 12 [25], the different rates of changes in the neuromuscular and musculotendinous systems before PHV are likely to lead to a phenomenon known as adolescent awkwardness [26,27] near the time of PHV. Similar to our findings, previous data demonstrated that a period of a slower performance development exists approximately 1.5 to 2.5 years before PHV in male pre-adolescents, representing the chronological age of 13 or 14 years [13,28]. Moreover, the relative jumping height normalized to the person’s height shows minimal alterations during this period [13,25]. Furthermore, these data are consistent with previous reports [29,30], which demonstrated that boys jump higher than girls, especially when they are at the same stage of maturation. A previous study showed that male adolescents exhibited greater jumping capability than females from the age of 11 onwards, while females’ capacity did not change significantly between the ages of 12 and 13 [31]. The current findings corroborate the widely documented sex differences in vertical jumping performance and height during adolescence [31,32], reflecting the differing time courses and/or adaptations in tendon properties and the neuromuscular system between the sexes during maturation.

Concerning the alterations in tendon properties, our findings revealed a time-specific pattern of changes across the two sexes. In agreement with our findings, previous data showed that tendon stiffness is higher in male adolescents than in females [14,24]. This was found to be associated with improved muscle–tendon unit capacity, a reduction in electromechanical delay, an increased rate of torque development, and consequently, increased jumping performance [33]. On the other hand, compliant tendons could be beneficial for storing more elastic energy during an SSC exercise, but it is less efficient to use this stored energy during the shortening phase [34]. The present study showed that male adolescents undergo increased AT stiffness from 18 to 9 months before PHV, while this occurs in female adolescents only near PHV. These results indicate that boys’ alterations may be related to the changes that occur through testosterone secretion [35,36]. The increases in testosterone levels lead to increased muscle mass and strength capacity. In contrast, the long-term changes in females due to estrogen secretion do not affect lean muscle mass and strength capacity. Given that tendon stiffness presents different rates of change and absolute values, the two sexes possibly adopt different strategies to optimize their performance during SSC [37].

A significant time effect was found in eccentric and concentric MG and TA activity in boys, but not in girls. Specifically, boys underwent increased MG activity during CMJ, near PHV, simultaneously with a decrease in TA activity at the same time point. It is known that muscle architecture changes throughout the nonlinear process of adolescent maturation [7]. Radnor et al. (2020), in a recent study, found that the MG muscle architecture influences force production in explosive-type exercises, mainly after PHV [1]. Combining these results with those of the present study we can come to the conclusion that, despite the increase in MG activity during CMJ at PHV, jump height would probably increase after PHV in boys, as a consequence of maturity adaptations. Furthermore, the values of antagonist activity and eccentric co-contraction decreased near PHV in boys. Ford et al. (2008), in a review article, concluded that the inhibition of antagonist muscles combined with decreased co-contraction is related to increased power output at joints and, as a consequence, efficient adaptation in SSC activities [38]. Lower activity of TA and lower co-contraction, accompanied by higher performance, was found by Lazaridis et al. (2010) in adult men compared to pre-adolescent boys in a drop jump [9]. It seems that neuromuscular adaptations that happen in the maturity process affect SSC activities, with a jump height improvement apparent after PHV in boys.

Meanwhile, females revealed a more stable low increase in AT stiffness accompanied by increased co-contraction, compared to boys, around the ankle joint. Increased co-contraction is associated with increased ankle stiffness [39,40], which is the mechanism employed to overcome reduced tendon stiffness and promote joint stabilization [41]. Although the ankle joint becomes stiffer, leading to better storage of elastic energy, this alteration could also decrease the mechanical work produced during plantar flexion in the final phase of the vertical jump [42]. Additionally, it could result in lower angular velocity and a reduced mechanical moment during the concentric phase, leading to less efficient performance [40]. Prior research indicated that higher agonist–antagonist co-contraction explains the lower levels of strength and power between children and adults in multijoint contractions [43]. Consequently, the more immature neuromuscular system in girls during the growth spurt, concerning SSC activities, seems to affect their CMJ performance. The different neuromuscular adaptations to adolescent maturation include peripheral regulation in vertical jumping for boys, and an immature system for girls.

The present study is not without limitations. It is regrettable that the activity of the knee flexors and the soleus were not evaluated during the CMJ. Furthermore, the process of CMJ performance was not assessed in the time period after the PHV. Additionally, it was not feasible within the scope of this study to examine the contribution of training to CMJ performance for each participant.

## 5. Conclusions

The present findings suggest that male and female adolescents adopt distinct neuromuscular activation strategies related to the level of tendon stiffness. Male adolescents show higher agonist and lower antagonist muscle activation, which lead to higher force production, greater tendon strain, and better use of elastic energy. In contrast, females show greater co-contraction, as a mechanism to increase joint stiffness and optimize SSC performance. These differences in activation strategies between genders underline the importance of considering different approaches in training to maximize performance and reduce injury risk in adolescent athletes. However, no significant changes in jumping ability were observed over time, showing that the maturation process does not affect SSC performance. Future studies should examine the mechanism of vertical jumping in adolescents after the critical period of PHV.

## Figures and Tables

**Table 1:** Between-subjects effects (sex differences).

DV	F	Sig	Partial η^2^
height	13.358	<0.001 **	0.271
relheight	7.112	0.011 *	0.165
AT stiffness	58.307	<0.001 **	0.618
eccRMSMG	4.723	0.036 *	0.116
conRMSMG	4.375	0.044 *	0.108
eccRMSTA	4.524	0.040 *	0.112
conRMSTA	0.942	0.338	0.026
ecccoRMS	0.440	0.511	0.012
concoRMS	6.145	0.018 *	0.146

* *p* < 0.05, ** *p* < 0.001.

## Data Availability

The original contributions presented in this study are included in the article. Further inquiries can be directed to the corresponding author.

## References

[1] Radnor J.M., Oliver J.L., Waugh C.M., Myer G.D., Lloyd R.S. (2020). The Influence of Maturity Status on Muscle Architecture in School-Aged Boys. Pediatr. Exerc. Sci..

[2] Kubo K., Teshima T., Hirose N., Tsunoda N. (2014). Growth Changes in Morphological and Mechanical Properties of Human Patellar Tendon In Vivo. J. Appl. Biomech..

[3] Pentidis N., Mersmann F., Bohm S., Giannakou E., Aggelousis N., Arampatzis A. (2020). Effects of Long-Term Athletic Training on Muscle Morphology and Tendon Stiffness in Preadolescence: Association with Jump Performance. Eur. J. Appl. Physiol..

[4] Grosset J.F., Mora I., Lambertz D., Perot C. (2008). Voluntary Activation of the Triceps Surae in Prepubertal Children. J. Electromyogr. Kinesiol..

[5] Kubo K., Kanehisa H., Kawakami Y., Fukanaga T. (2001). Growth Changes in the Elastic Properties of Human Tendon Structures. Int. J. Sports Med..

[6] Waugh C.M., Korff T., Fath F., Blazevich A.J. (2014). Effects of Resistance Training on Tendon Mechanical Properties and Rapid Force Production in Prepubertal Children. J. Appl. Physiol..

[7] Radnor J.M., Oliver J.L., Waugh C.M., Myer G.D., Moore I.S., Lloyd R.S. (2018). The Influence of Growth and Maturation on Stretch-Shortening Cycle Function in Youth. Sport Med..

[8] Piponnier E., Martin V., Bourdier P., Biancarelli B., Kluka V., Garcia-Vicencio S., Jegu A.-G., Cardenoux C., Morio C., Coudeyre E. (2019). Maturation-Related Changes in the Development and Etiology of Neuromuscular Fatigue. Eur. J. Appl. Physiol..

[9] Lazaridis S., Bassa E., Patikas D., Giakas G., Gollhofer A., Kotzamanidis C. (2010). Neuromuscular Differences between Prepubescents Boys and Adult Men during Drop Jump. Eur. J. Appl. Physiol..

[10] Lloyd R.S., Oliver J.L., Hughes M.G., Williams C.A. (2011). The Influence of Chronological Age on Periods of Accelerated Adaptation of Stretch-Shortening Cycle Performance in Pre and Postpubescent Boys. J. Strength Cond. Res..

[11] Bobbert M.F., Van Soest A.J. (2001). Why Do People Jump the Way They Do?. Exerc. Sport Sci. Rev..

[12] Secomb J., Lundgren L., Farley O., Tran T., Nimphius S., Sheppard J. (2015). Relationships Between Lower-Body Muscle Structure and Lower-Body Strength, Power, and Muscle-Tendon Complex Stiffness. J. Strength Cond. Res..

[13] Wdowski M.M., Noon M., Mundy P.D., Gittoes M.J.R., Duncan M.J. (2020). The Kinematic and Kinetic Development of Sprinting and Countermovement Jump Performance in Boys. Front. Bioeng. Biotechnol..

[14] Chalatzoglidis G., Blazevich A.J., Arabatzi F. (2012). An Adaptive Model of Achilles Tendon Mechanical Properties during Adolescence: Effect of Sex. Muscles Ligaments Tendons J. MLTJ.

[15] Neugebauer J.M., Hawkins D.A. (2012). Identifying Factors Related to Achilles Tendon Stress, Strain, and Stiffness before and after 6 Months of Growth in Youth 10–14 Years of Age. J. Biomech..

[16] Kozinc Ž., Žitnik J., Smajla D., Šarabon N. (2022). The Difference between Squat Jump and Countermovement Jump in 770 Male and Female Participants from Different Sports. Eur. J. Sport. Sci..

[17] Tanner J.M. (1981). Growth and Maturation during Adolescence. Nutr. Rev..

[18] Mirwald R.L., Baxter-Jones A.D.G., Bailey D.A., Beunen G.P. (2002). An Assessment of Maturity from Anthropometric Measurements. Med. Sci. Sports Exerc..

[19] Padulo J., Oliva F., Frizziero A., Maffulli N. (2016). Muscles, Ligaments and Tendons Journal—Basic Principles and Recommendations in Clinical and Field Science Research: 2016 Update. Muscles. Ligaments Tendons J..

[20] SENIAM Surface Electromyography for the Non-Invasive Assessment of Muscles. http://www.seniam.org.

[21] Maganaris C.N., Baltzopoulos V., Sargeant A.J. (2000). In Vivo Measurement-Based Estimations of the Human Achilles Tendon Moment Arm. Eur. J. Appl. Physiol..

[22] Arampatzis A., De Monte G., Karamanidis K. (2008). Effect of Joint Rotation Correction When Measuring Elongation of the Gastrocnemius Medialis Tendon and Aponeurosis. J. Electromyogr. Kinesiol..

[23] Mademli L., Arampatzis A., Morey-Klapsing G., Brüggemann G.P., Bruggemann G.P., Brüggemann G.P. (2004). Effect of Ankle Joint Position and Electrode Placement on the Estimation of the Antagonistic Moment during Maximal Plantarflexion. J. Electromyogr. Kinesiol..

[24] Waugh C.M., Blazevich A.J., Fath F., Korff T. (2012). Age-Related Changes in Mechanical Properties of the Achilles Tendon. J. Anat..

[25] Focke A., Strutzenberger G., Jekauc D., Worth A., Woll A., Schwameder H. (2013). Effects of Age, Sex and Activity Level on Counter-Movement Jump Performance in Children and Adolescents. Eur. J. Sport Sci..

[26] Meyers R.W., Oliver J.L., Hughes M.G., Lloyd R.S., Cronin J.B. (2016). The Influence of Maturation on Sprint Performance in Boys over a 21-Month Period. Med. Sci. Sport Exerc..

[27] Philippaerts R.M., Vaeyens R., Janssens M., Van Renterghem B., Matthys D., Craen R., Bourgois J., Vrijens J., Beunen G., Malina R.M. (2006). The Relationship between Peak Height Velocity and Physical Performance in Youth Soccer Players. J. Sport Sci..

[28] Meyers R.W., Oliver J.L., Hughes M.G., Cronin J.B., Lloyd R.S. (2015). Maximal Sprint Speed in Boys of Increasing Maturity. Pediatr. Exerc. Sci..

[29] Klausen K., Schibye B., Rasmussen B. (1989). A Longitudinal Study of Changes in Physical Performance of 10- to 15-Year-Old Girls and Boys. Children and Exercise. Champaign Hum. Kinet..

[30] Temfemo A., Hugues J., Chardon K., Mandengue S.-H., Ahmaidi S. (2009). Relationship between Vertical Jumping Performance and Anthropometric Characteristics during Growth in Boys and Girls. Eur. J. Pediatr..

[31] Taylor M.J., Cohen D., Voss C., Sandercock G.R. (2009). Vertical Jumping and Leg Power Normative Data for English School Children Aged 10--15 Years. J. Sports Sci..

[32] Negra Y., Sammoud S., Myers T., Nevill A.M., Chaabene H. (2024). Normative Values for Measures of Physical Fitness Among Tunisian School Children. J. Sci. Sport Exerc..

[33] Mersmann F., Charcharis G., Bohm S., Arampatzis A. (2017). Muscle and Tendon Adaptation in Adolescence: Elite Volleyball Athletes Compared to Untrained Boys and Girls. Front. Physiol..

[34] Kubo K., Kawakami Y., Fukunaga T. (1999). Influence of Elastic Properties of Tendon Structures on Jump Performance in Humans. J. Appl. Physiol..

[35] Hansen M., Kjaer M. (2014). Influence of Sex and Estrogen on Musculotendinous Protein Turnover at Rest and after Exercise. Exerc. Sport Sci. Rev..

[36] Vingren J.L., Kraemer W.J., Ratamess N.A., Anderson J.M., Volek J.S., Maresh C.M. (2012). Testosterone Physiology in Resistance Exercise and Training: The up-Stream Regulatory Elements. Sports Med..

[37] Ford K.R., Myer G.D., Hewett T.E. (2010). Longitudinal Effects of Maturation on Lower Extremity Joint Stiffness in Adolescent Athletes. Am. J. Sports Med..

[38] Ford K.R., van den Bogert J., Myer G.D., Shapiro R., Hewett T.E. (2008). The Effects of Age and Skill Level on Knee Musculature Co-Contraction during Functional Activities: A Systematic Review. Br. J. Sports Med..

[39] Arampatzis A., Bruggemann G.P., Klapsing G.M. (2001). Leg Stiffness and Mechanical Energetic Processes during Jumping on a Sprung Surface. Med. Sci. Sport. Exerc..

[40] Márquez G., Morenilla L., Taube W., Fernández-del-Olmo M. (2014). Effect of Surface Stiffness on the Neural Control of Stretch-Shortening Cycle Movements. Acta Physiol..

[41] Mengarelli A., Maranesi E., Burattini L., Fioretti S., Di Nardo F. (2017). Co-Contraction Activity of Ankle Muscles during Walking: A Gender Comparison. Biomed. Signal Process. Control.

[42] Hof A.L., Van Zandwijk J.P., Bobbert M.F. (2002). Mechanics of Human Triceps Surae Muscle in Walking, Running and Jumping. Acta Physiol. Scand..

[43] Dotan R., Mitchell C., Cohen R., Klentrou P., Gabriel D., Falk B. (2012). Child-Adult Differences in Muscle Activation—A Review. Pediatr. Exerc. Sci..

